# Editorial: Sharing Economy and the Issue of (Dis)Trust

**DOI:** 10.3389/fpsyg.2021.689722

**Published:** 2021-05-28

**Authors:** Eva Hofmann, Barbara Hartl, Ann-Marie Ingrid Nienaber

**Affiliations:** ^1^Institute of Psychology, University of Graz, Graz, Austria; ^2^Institute for Marketing and Consumer Research, WU Vienna University of Economics and Business, Vienna, Austria; ^3^Centre for Trust, Peace and Social Relations, Coventry University, Coventry, United Kingdom

**Keywords:** trust, distrust, technology, sharing economy, sharing behavior

## Introduction

Economy has changed; while in earlier times the possession of goods has been important to consumers, nowadays consumers rather try to have access to them (Belk, 2014). The so-called sharing economy enables consumers to share goods and services, such as rooms (cf. Airbnb), mobility services (cf. Blablacar), or self-produced vegetables (cf. community gardens). The Internet was key to the development of the sharing economy; with this achievement, the sharing economy expanded (Puschmann and Alt, 2016), and research on the sharing economy accumulated.

Trust is a fundamental aspect that keeps the sharing economy running (Möhlmann, 2015). Trust means that consumers make themselves vulnerable toward different actors (Rousseau et al., 1998). There needs to be trust toward the sharing organization (e.g., Airbnb), toward consumers offering their goods and services, toward other consumers and also toward the technology providing the platform. Several articles (e.g., Kong et al., 2020) address the important issue of trust in the sharing economy, but the more or less antagonist of trust, i.e., distrust, has not been addressed in research so far. Distrust is often seen as an individual's unwillingness to accept vulnerability to the actions of other people based on pervasive negative perceptions and expectations of the other people's motives, intentions, or behaviors (Bijlsma-Frankema et al., 2015). The relevance of distrust often resonates implicitly in the publications on trust in the sharing economy (e.g., Möhlmann, 2015), but was not explicitly addressed in previous research. For that reason, the current Research Topic opens up a forum, in which research not only on trust but also on distrust in the sharing economy can gain center stage.

## Overview of Articles in the Research Topic

The first article of the Research Topic by Gruber focuses on the formation of trust in the sharing economy. The paper distinguishes between trust formation in the community-based economy, using the example of community supported agriculture (CSA), and the platform economy, illustrated with the platform Airbnb. Trust formation in both institutions immensely differs; while in CSA trust is built through personal relations and face-to-face communication in small groups, trust is achieved via technological innovation in form of applications on Airbnb's website. The paper not only intends to present the different kinds of trust formation, but also presents models and hypotheses to enhance (dis)trust research in the sharing economy.

The second paper was written by Marth et al. and investigates the impact of regulation in the sharing economy on consumers' willingness to take the risk of participating in a car-sharing community with trust being a mediator between regulation and the risk preference. The authors distinguish between soft and harsh regulation and between implicit and reason-based trust on a vertical vs. horizontal level. A laboratory and an online experiment reveal that the combination of high soft and low harsh regulation increases consumers' willingness to take part in a car-sharing community, whereby this relationship is mediated by vertical and horizontal reason-based trust. Thus, trust can be generated by soft regulation and leads to participation in the sharing economy.

The third article by Bhappu et al. covers an uncommon area of the sharing economy; the paper focuses on a platform, which is used by work colleagues but not strangers to share goods and services. The platform is sponsored by the co-workers' employer. By means of interviews, the authors investigate how trust is built and generate a model that indicates how the aspects identity, interaction, and issue frames are responsible for the beliefs of employees regarding the trustworthiness of their co-workers and subsequently their sharing intention.

The fourth paper is covering specifically the issue of distrust. Based on empirical data collected over the period of 3 years via workshops and semi-structured interviews with seven public authorities in Europe, Nienaber et al. demonstrate that the “real” obstacle for not sharing data lies in the hands of the public authority's servants of the middle and high-level management. They show firstly, that distrust in a sharing economy can be perceived toward four different referents and thus, needs to be specifically addressed during relationship building. Secondly, they provide evidence on the idea of a self-amplifying circle of distrust driven by negative reciprocity. When distrust reached its threshold, the results demonstrate that distrust is able to take an all-encompassing state. This prohibits any kind of sharing economy.

## Conclusion

This Research Topic is a fine collection of articles on trust in the sharing economy, but specifically it is covering the important issue of distrust. This is exceptional and opens up a lot of room for more in-depth research answering questions such as: How can distrust in the sharing economy be reduced or overcome to enhance the likelihood of consumers to take part in a sharing economy? What are the triggers of distrust? How can public authorities provide organizational support to foster participation in sharing economies by drafting legislation that protects consumers and thereby diminishes distrust? These are only few questions that need to be answered to enable sharing on a large scale. With the current Research Topic we have built the starting point for such an endeavor.

## Author Contributions

EH wrote the editorial. BH and A-MN reworked the editorial. All authors contributed to the article and approved the submitted version.

## Conflict of Interest

The authors declare that the research was conducted in the absence of any commercial or financial relationships that could be construed as a potential conflict of interest.
